# Cardiac biomarkers are associated with maximum stage of acute kidney injury in critically ill patients: a prospective analysis

**DOI:** 10.1186/s13054-017-1674-5

**Published:** 2017-04-12

**Authors:** Ryan Haines, Siobhan Crichton, Jessica Wilson, David Treacher, Marlies Ostermann

**Affiliations:** 1 Department of Critical Care, King’s College London, Guy’s and St Thomas’ Foundation Hospital, London, SE1 7EH UK; 2 Division of Health and Social Care Research, King’s College London, London, UK

**Keywords:** Acute kidney injury, Renal replacement therapy, Brain natriuretic peptide, Troponin, Biomarker

## Abstract

**Background:**

This study aimed to investigate whether cardiac troponin T (cTnT), cardiac troponin I (cTnI) and serum N-terminal pro-brain natriuretic peptide (NT-proBNP) are associated with acute kidney injury (AKI) and need for acute renal replacement therapy (RRT) in adult patients admitted to the intensive care unit (ICU).

**Methods:**

We analysed prospectively collected data for patients admitted to the ICU between June and December 2010 for non-cardiac reasons. The Kidney Disease Improving Global Outcomes creatinine criteria were applied to identify patients with AKI including those who received acute RRT. Severity of illness was determined by the Acute Physiology and Chronic Health Evaluation (APACHE) II score and the Serial Organ Failure Assessment (SOFA) score. Regression analyses were performed to assess the association between cTnT, cTnI and NT-proBNP concentrations on the first day of ICU stay, maximum AKI stages and need for acute RRT. Sensitivity analysis was performed in which patients who developed a myocardial infarction during their stay in the ICU were excluded.

**Results:**

Of 138 patients included, 73 (53%) had AKI and 40 (29%) required acute RRT. Patients with AKI were significantly older, more likely to have sepsis and had higher APACHE II and SOFA scores on admission to the ICU. In univariable analysis, cTnT, cTnI and NT-proBNP were significantly higher in those with AKI requiring acute RRT, but after adjustment for baseline differences in severity of illness, cumulative fluid balance and pre-existing comorbidities, only NT-proBNP remained significantly associated with worst stage of AKI and need for RRT. cTnT and cTnI were independently associated with the odds of any AKI but not with need for RRT.

In a sensitivity analysis in which patients who had an acute myocardial infarction while in the ICU were excluded, NT-proBNP remained independently associated with AKI and acute RRT.

**Conclusions:**

In critically ill patients admitted to the ICU for non-cardiac reasons, admission NT-proBNP had the strongest independent association with maximum stage of AKI and need for RRT.

**Electronic supplementary material:**

The online version of this article (doi:10.1186/s13054-017-1674-5) contains supplementary material, which is available to authorized users.

## Background

Acute kidney injury (AKI) is a serious complication of critical illness that affects more than 50% of patients admitted to the intensive care unit (ICU) [1, 2]. AKI is independently associated with short and long-term complications, mortality and increased healthcare costs [3]. There is an urgent need to identify patients who are at risk of developing AKI in order to implement renoprotective strategies and avoid nephrotoxic exposures. Numerous novel biomarkers for AKI have been tested in critically ill patients. Although several markers have been found to indicate the onset of AKI before serum creatinine increases, none has been integrated into routine clinical practice [4].

A few studies have explored the role of cardiac biomarkers in predicting AKI, the rationale being that cardiac and renal function are closely linked (often referred to as cardio-renal syndrome [5]). For instance, Thiengo et al. [6] studied 29 ICU patients with incident sepsis and concluded that cardiac troponin I (cTnI) on admission predicted the development of AKI and the need for acute renal replacement therapy (RRT). Similarly, a study in 34 consecutive ICU patients showed that patients with AKI on presentation or during the ICU stay had significantly higher levels of the cardiac biomarker brain natriuretic peptide (BNP) relative to patients without AKI [7].

These studies were relatively small, and larger studies are required to confirm the findings. Our aim was to explore the relationship between the cardiac biomarkers cTnI, cardiac troponin T (cTnT) and serum N-Terminal pro-brain natriuretic peptide (NT-proBNP) and the development of AKI and the need for RRT in a larger patient cohort than previously undertaken.

## Methods

We performed a sub-analysis of data that were collected previously for a prospective observational study aimed at exploring the epidemiology of troponin elevation in critically ill patients admitted to the ICU for non-cardiac reasons [8].

### Setting

Guy’s and St Thomas’ NHS Foundation Hospital is a tertiary-care centre with a 43-bed level 3 multidisciplinary adult ICU. The ICU has a fully computerized electronic patient record system in which all data are recorded at the time of generation.

### Patient population

As described previously [8], between June and December 2010 we enrolled consecutive adult patients (≥18 years) who had been admitted to the ICU for non-cardiac reasons. Patients with a high probability of cardiac injury or a primary cardiac diagnosis at ICU admission were excluded, specifically those with a clinical diagnosis of myocardial infarction (MI) or out-of-hospital cardiac arrest, patients who were post cardiac surgery or cardiac intervention and patients admitted following thoracic trauma with a high likelihood of myocardial injury. Patients were also excluded if they had been transferred from another ICU, had previously been admitted to the ICU during the same hospital stay or were expected to remain in the ICU for <48 hours.

Patients were recruited within 36 hours of admission to the ICU and blood was taken for measurement of cTnT, cTnI and NT-proBNP. The blood samples were stored at –70 °C until batch analysis at the end of the study. For the purpose of this analysis, patients with pre-existing end-stage renal failure (ESRF) were excluded.

### Laboratory analysis

cTnT was measured using the Roche electrochemiluminescent high-sensitivity sandwich immunoassay on the Elecsys 2010. The quoted analytical range was 3–10,000 ng/L, total coefficients of variation (CVs) were 1.5–3.4% (measured between 24 and 2665 ng/L) and the reference range was <15 ng/L (99th percentile).

cTnI was measured using the Siemens Tnl-Ultra three-site sandwich immunoassay on the Advia Centaur. The quoted analytical range was 6–50,000 ng/L, total CVs were 2.7–5.3% (measured between 80 and 27,200 ng/L) and the reference range was <39 ng/L.

NT-proBNP was measured using the Diagnostic Products Corporation Immulite 2500 chemiluminescent sandwich immunoassay. The analytical range was 20–35,000 pg/ml, the quoted total CVs were 3.4–5.6% (measured between 40.9 and 32,096 pg/ml) and reference range was <125 pg/ml in patients <75 years old and <450 pg/ml in those >75 years old.

### Data collection

We collected demographics, admission diagnosis, cardiovascular risk factors (ischaemic heart disease (IHD), diabetes, hypertension, any type of vascular disease), Acute Physiology and Chronic Health Evaluation (APACHE) II score, Sequential Organ Failure Assessment (SOFA) score, serum creatinine, presence of sepsis and ICU and hospital outcome. Cumulative fluid balance in the first 24 hours in the ICU was determined from all recorded fluid input and output data.

AKI was defined by the creatinine criteria of the Kidney Disease Improving Global Outcomes (KDIGO) classification [9, 10]. We recorded the maximum AKI stage during ICU admission. The lowest serum creatinine concentration within the previous 6 months was used as baseline. If a baseline serum creatinine was not available, we estimated it by back-calculation using the Modification of Diet in Renal Disease (MDRD) formula for an estimated glomerular filtration rate (eGFR) of 75 ml/min/1.73 m^2^ [10]. If the patient was known to have pre-existing chronic kidney disease (CKD) but a previous serum creatinine result was not available, baseline serum creatinine was again back-calculated for the relevant stage of CKD using the MDRD formula as already described. Treatment with RRT was recorded.

We also recorded whether patients had developed an acute MI during ICU admission. The diagnosis of MI was based on an elevated cTnT ≥ 15 ng/L and contemporaneous ischaemic ECG changes according to the most recent consensus criteria of the European Society of Cardiology and American College of Cardiology [11].

### Statistics

Continuous data were summarized as the mean (standard deviation (SD)) or the median (interquartile range (IQR)) where the data were skewed, and were compared between patients who did and did not develop AKI using the *t* test or Mann–Whitney *U* test, as appropriate. Categorical data were summarized as frequency (percentage) and compared using the chi-square test.

The AKI stage was defined as the maximum AKI stage during admission. The associations between cardiac troponin concentrations and odds of AKI were explored using logistic regression models with development of AKI defined in three different ways. The first set of models were used to model odds of any AKI versus none, the second set looked at stage 3 AKI versus lower stage or no AKI and the third set modelled the odds of need for RRT versus lower stage or no AKI. Troponin concentrations were highly skewed and therefore were log_2_ transformed prior to inclusion in the models to meet the assumption of a linear relationship between troponin level and the log odds. Results are therefore expressed as an odds ratio (OR) which represents the change in odds for a doubling of troponin level.

Multivariable logistic regression models were used to adjust for baseline characteristics, with the number of covariates included in the model reduced using principal components analysis. Because of strong correlation between cTnI and cTnT, we analysed cTnI, cTnT and NT-proBNP levels in separate models. A sensitivity analysis was carried out in which patients who developed an MI during their stay in the ICU before or after the development of AKI were excluded. A second sensitivity analysis was performed in which patients with any degree of AKI on admission to ICU were excluded. The statistical analyses were carried out using Stata 14 MP.

## Results

Of 144 patients recruited in the original study [8], six were excluded due to pre-existing ESRF. As a result 138 patients were included in the analysis, of whom 73 (53%) developed AKI. Thirteen patients (9.4%) had maximum AKI stage 1, 12 patients (8.7%) had AKI stage 2 and 48 patients (35%) had AKI stage 3, of whom 40 were treated with acute RRT. The median time between the day of admission to the ICU and the day of the worst AKI stage was 3 days.

Patients who developed AKI were significantly older, had higher APACHE II and SOFA scores on admission to the ICU, were more likely to have sepsis and had higher serum bilirubin and lactate concentrations than those without AKI (Table 1).

**Table 1:** Patient characteristics and outcomes

Parameter	All (*n* = 138)	No AKI (*n* = 65)	Any AKI (*n* = 73)	*p* value
Baseline characteristics				
Age, median (IQR)	65.5 (49–76)	61 (41–72)	69 (54–77)	0.006
Male gender, *n* (%)	80 (58.0)	41 (63.1)	39 (53.4)	0.252
Comorbidities				
IHD, *n* (%)	22 (15.9)	11 (16.9)	11 (15.1)	0.766
Hypertension, *n* (%)	49 (35.5)	21 (32.3)	28 (38.4)	0.459
Admission diagnosis category, *n* (%)				
Acute kidney injury	8 (5.8)	–	8 (11.0)	
Cardiac emergency	6 (4.3)	3 (4.6)	3 (4.1)	
Gastrointestinal emergency	13 (9.4)	8 (12.3)	5 (6.8)	
Liver failure	3 (2.1)	–	3 (4.1)	
Metabolic emergency	4 (2.9)	–	4 (5.5)	
Neurological emergency/overdose	13 (9.4)	12 (18.5)	1 (1.4)	
Post surgery	25 (18.1)	13 (20)	12 (16.4)	
Respiratory failure	27 (19.6)	17 (26.1)	10 (13.7)	
Sepsis	39 (28.3)	12 (18.5)	27 (37.0)	
Parameters on admission to ICU				
APACHE II, mean (SD)	19.4 (6.3)	16.2 (5.1)	22.2 (5.9)	<0.001
SOFA score, mean (SD)	7.5 (3.7)	6.0 (3.0)	8.9 (3.8)	<0.001
Lactate (μmol/L), median (IQR)	1.7 (1.1–3.3)	1.4 (0.9–2.1)	1.9 (1.2–4.5)	0.005
Bilirubin (μmol/L), median (IQR)	10 (7–19)	10 (6–14)	12 (7–30)	0.031
Sepsis, *n* (%)	39 (28.3)	12 (18.5)	27 (37.0)	0.016
Parameters in first 24 hours in ICU				
Cumulative fluid balance (ml), median (IQR)	3196 (1606–5166)	2561 (1088–4023)	3730 (2316–5800)	0.002
No AKI, *n* (%)	77 (55.8)	–	77 (55.8)	
Any stage of AKI, *n* (%)				
1	14 (10.1)	–	14 (10.1)	
2	14 (10.1)	–	14 (10.1)	
3 without RRT	5 (3.6)	–	5 (3.6)	
3 with RRT	28 (20.3)	–	28 (20.3)	
Outcomes				
MI (in ICU), *n* (%)	20 (14.5)	9 (13.9)	11 (15.1)	0.839
Maximum stage of AKI stage during stay in ICU, *n* (%)				
1	13 (9.4)	–	13 (9.4)	
2	12 (8.7)	–	12 (8.7)	
3 without RRT	8 (5.8)	–	8 (5.8)	
3 with RRT	40 (29.0)	–	40 (29.0)	

*APACHE* Acute Physiology and Chronic Health Evaluation, *AKI* acute kidney injury, *ICU* intensive care unit, *IHD* ischaemic heart disease, *IQR* interquartile range, *MI* myocardial infarction, *RRT* renal replacement therapy, *SD* standard deviation, *SOFA* Sequential Organ Failure Assessment

### Univariable and multivariable analyses

In univariable analysis, cTnI, cTnT and NT-proBNP concentrations within the first 36 hours of ICU admission were significantly higher in patients who developed AKI or required RRT, with the odds of AKI increasing as troponin increased (all *p* ≤ 0.001) (Table 2).

**Table 2:** Associations between cardiac troponin I, cardiac troponin T and NT-proBNP and maximum stage of AKI

Cardiac biomarker	Median (IQR),	Median (IQR),	Unadjusted OR^a^ (95% CI)	*p* value	Adjusted^b^ OR^a^ (95% CI)	Adjusted^a^ *p* value
	Group 1	Group 2				
Cardiac troponin I						
Any AKI (1) vs no AKI (2)	0.14 (0.04–0.70)	0.03 (0.02–1.0)	1.37 (1.16–1.62)	<0.001	1.20 (1.00–1.45)	0.044
Stage 3 AKI (1) vs lower stage (2)	0.22 (0.06 –1.03)	0.04 (0.02–0.16)	1.33 (1.14–1.54)	<0.001	1.17 (0.98–1.41)	0.085
Stage 3 AKI with RRT (1) vs lower stage (2)	0.27 (0.06–1.03)	0.04 (0.02–0.19)	1.27 (1.10–1.40)	0.001	1.09 (0.90–1.32)	0.376
Cardiac troponin T						
Any AKI (1) vs no AKI (2)	0.07 (0.04–0.16)	0.02 (0.01–0.05)	1.63 (1.32–2.01)	<0.001	1.36 (1.06–1.74)	0.014
Stage 3 AKI (1) vs lower stage (2)	0.10 (0.05–0.21)	0.03 (0.01–0.07)	1.58 (1.28–1.96)	<0.001	1.40 (1.06–1.84)	0.018
Stage 3 AKI with RRT (1) vs lower stage (2)	0.10 (0.05–0.21)	0.03 (0.01–0.08)	1.49 (1.21–1.83)	<0.001	1.27 (0.95–1.69)	0.104
NT-proBNP						
Any AKI (1) vs no AKI (2)	8888 (2504–18685)	1543 (239–5301)	1.41 (1.22–1.64)	<0.001	1.23 (1.02–1.47)	0.027
Stage 3 AKI (1) vs lower stage (2)	10879 (2792–19797)	1609 (480–7055)	1.52 (1.27–1.82)	<0.001	1.44 (1.14–1.82)	0.002
Stage 3 AKI with RRT (1) vs lower stage (2)	11302 (2792–21512)	1718 (531–7293)	1.48 (1.22–1.78)	<0.001	1.40 (1.09–1.81)	0.008

*NT-proBNP* N-terminal pro-brain natriuretic peptide, *AKI* acute kidney injury, *RRT* renal replacement therapy, *IQR* interquartile range, *CI* confidence interval, *OR* odds ratio, *APACHE* Acute Physiology and Chronic Health Evaluation

^a^ORs estimated using logistic regression models with troponin levels log_2_ transformed. ORs represent the change in odds of AKI associated with a doubling of troponin levels

^b^Adjusted for age, APACHE II score, gender, hypertension, diabetes, ischaemic heart disease, sepsis, lactate, bilirubin and cumulative fluid balance in the first 24 hours in the ICU (reduced to three principal components in models)

After adjustment for age, APACHE II score, gender, hypertension, diabetes, IHD, sepsis, serum lactate, serum bilirubin concentrations and cumulative fluid balance in the first 24 hours in the ICU, NT-proBNP remained independently associated with odds of worst AKI stage and need for RRT (Table 2). cTnT was independently associated with an increased odds of any AKI, as opposed to none, and the development of maximum AKI stage 3 as opposed to a lower AKI stage, but there was no independent association with need for RRT. cTnI was independently associated with an increased odds of any AKI but there was no association with development of AKI stage 3 as opposed to a lower AKI stage.

### Sensitivity analyses

Sensitivity analysis showed that NT-proBNP and cTnT remained independent risk factors for AKI even when patients who had a MI while in the ICU were excluded (Table 3). NT-proBNP was also independently associated with the need for RRT.

**Table 3:** Associations between cardiac troponin I, cardiac troponin T and NT-proBNP and odds of maximum stage of AKI – excluding patients with confirmed MI

Parameter	Number of patients		Median (IQR)	Median (IQR)	OR^a^ (95% CI)	*p* value	Adjusted^b^ OR^a^ (95% CI)	Adjusted^b^ *p* value
	(1)	(2)	(1)	(2)				
Cardiac troponin I								
Any AKI (1) vs no AKI (2)	62	56	0.12 (0.04–0.69)	0.02 (0.02–0.06)	1.50 (1.22–1.85)	<0.001	1.31 (1.05–1.63)	0.015
Stage 3 AKI (1) vs lower stage (2)	40	78	0.17 (0.05–1.03)	0.03 (0.02–0.08)	1.41 (1.19–1.68)	<0.001	1.25 (1.01–1.56)	0.043
Stage 3 AKI with RRT (1) vs lower stage (2)	34	84	0.17 (0.05–1.08)	0.04 (0.02–0.12)	1.35 (1.14–1.60)	0.001	1.12 (0.93–1.45)	0.199
Cardiac troponin T								
Any AKI (1) vs no AKI (2)	62	56	0.07 (0.03–0.15)	0.02 (0.01–0.04)	1.83 (1.42–2.35)	<0.001	1.55 (1.16–2.07)	0.003
Stage 3 AKI (1) vs lower stage (2)	40	78	0.08 (0.04–0.19)	0.03 (0.01–0.06	1.69 (1.32–2.15)	<0.001	1.51 (1.10–2.09)	0.012
Stage 3 AKI with RRT (1) vs lower stage (2)	34	84	0.08 (0.05–0.18)	0.03 (0.01–0.07)	1.55 (1.23–1.96)	<0.001	1.31 (0.94–1.84)	0.109
NT-proBNP								
Any AKI (1) vs no AKI (2)	62	56	7182 (2314–15,550)	1055 (192–2944)	1.47 (1.24–1.74)	<0.001	1.31 (1.06–1.61)	0.012
Stage 3 AKI (1) vs lower stage (2)	40	78	9179 (2561–16,179)	1609 (320–6015)	1.49 (1.23–1.81)	<0.001	1.45 (1.12–1.86)	0.004
Stage 3 AKI with RRT (1) vs lower stage (2)	34	84	10,105 (2617–17,069)	1681 (490–6416)	1.48 (1.21–1.81)	<0.001	1.43 (1.09–1.88)	0.011

*AKI* acute kidney injury, *RRT* renal replacement therapy, *IQR* interquartile range, *CI* confidence interval, *MI* myocardial infarction, *NT-proBNP* N-terminal pro-brain natriuretic peptide, *OR* odds ratio, *APACHE* Acute Physiology and Chronic Health Evaluation

^a^ORs estimated using logistic regression models with troponin levels log_2_ transformed. ORs represent the change in odds of AKI associated with a doubling of troponin levels

^b^Adjusted for age, APACHE II score, gender, hypertension, diabetes, ischaemic heart disease, sepsis, lactate, bilirubin and cumulative fluid balance in the first 24 hours in the ICU (reduced to three principal components in models)

Additional file 1 presents the results of a sensitivity analysis in which patients with any AKI on the day of admission to the ICU were excluded. Patients with AKI had significantly higher NT-proBNP concentrations.

## Discussion

The key finding of this study was that analysis were that in critically ill patients admitted for non-cardiac reasons, NT-proBNP on admission to the ICU was independently associated with the maximum stage of AKI, including the need for RRT. The independent association persisted after excluding patients who subsequently developed an acute MI during their stay in the ICU. cTnT was independently associated with AKI but not with need for RRT. cTnI was not an independent risk factor.

**Fig. 1:**
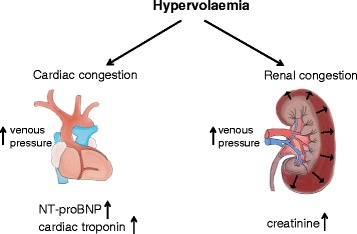
Association between cardiac and renal congestion. *NT-proBNP* N-terminal pro-brain natriuretic peptide

NT-proBNP had the strongest association with worst stage of AKI and need for RRT. NT-proBNP is a polypeptide secreted by the ventricles in response to excessive stretching of cardiomyocytes and its role is to promote natriuresis [12]. The link between ventricular dilatation, raised central venous pressure (CVP) and renal dysfunction is well established [13, 14] (Fig. 1). In patients with a raised CVP, the increased backward pressure propagates evenly in all districts of the venous system, including renal veins. As a result, renal congestion sets in and glomerular filtration and sodium excretion decrease [15–19]. Physiologically, it makes sense that NT-proBNP, a biomarker of ventricular dilatation, is associated with the development of AKI.

In patients with cardiac disease, BNP has been extensively investigated and found to have added predictive value for the development of AKI in patients with an ST-elevation MI or heart failure [20, 21]. BNP has also been described as a marker of the ‘cardio renal burden’ in patients admitted with a cardiac emergency and as a risk factor for the development of AKI after cardiac surgery [22–24].

To the best of our knowledge, the role of BNP as a biomarker for AKI in critically ill patients presenting without an acute cardiac emergency is limited to two studies. de cal et al. [7] conducted a prospective trial in 34 ICU patients admitted with a non-cardiac diagnosis and concluded that BNP levels predicted the development of AKI. However, there was no adjustment for age and severity of illness. Chou et al. [25] measured BNP on admission to the ICU and 24 hours later in 163 critically ill patients, and showed that changes in BNP between the day of admission and 24 hours later predicted the development of AKI, renal recovery and mortality. Again, there was no adjustment for other important risk factors, including severity of illness and underlying cardiac risk factors.

The role of BNP in sepsis has also been studied [26, 27]. Papanikolaou et al. [26] measured BNP concentrations in patients with sepsis and showed that BNP levels were raised in sepsis and septic shock, presumably as a result of pro-inflammatory cytokine release and biventricular dysfunction. However, the impact on renal function was not assessed.

To our best knowledge this is the largest study which demonstrates an independent association between NT-proBNP concentration on admission to the ICU and worst stage of AKI, including requirement for acute RRT. Our conclusions are strengthened by the fact that the association was maintained after exclusion of patients who developed an acute MI during their stay in ICU and after adjustment for cumulative fluid balance. If confirmed in future studies, NT-proBNP could serve as an easily available ‘alert’ for AKI and RRT in routine clinical practice.

However, it is important to acknowledge some potential limitations. As a single-centre retrospective study, the impact of unrecorded confounding factors cannot be excluded. This is particularly important when interpreting the association between NT-proBNP and acute RRT because there is no consensus about the optimal time of starting RRT and high variation in clinical practice. Second, we did not perform routine echocardiography in all patients to correlate the NT-proBNP results with ventricular dilatation and acknowledge that other mechanisms beyond ventricular stretch may stimulate NT-proBNP release [28]. Third, pre-existing creatinine results were only available for 54 patients (39%). In the remaining 84 patients (61%), baseline renal function was estimated by back-calculation using the MDRD formula. Although this method is supported by the KDIGO working group [9], we recognize that there are limitations, including misclassification of AKI [17]. Fourth, we defined sepsis according to the previous consensus criteria because our study was conducted before the new sepsis criteria were published [29]. Fifth, we calculated cumulative fluid balance in the first 24 hours in the ICU but did not include the fluid balance prior to ICU admission. Finally, the exact aetiology of AKI was not always documented by the treating clinical team. Despite these limitations, we believe that our findings are important and should prompt further research to verify our results.

## Conclusions

In patients without a cardiac diagnosis on admission to the ICU, admission NT-proBNP concentrations were independently associated with worst stage of AKI, including need for acute RRT. The cTnT and cTnI results on admission were associated with maximum stage of AKI but not with need for RRT. Larger studies are needed to evaluate the potential of NT-proBNP as a biomarker for AKI and RRT.

## Additional file

Additional file 1: Is a table presenting associations between cTnI, cTnT and NT-proBNP and the odds of AKI – excluding patients with any degree of AKI on day 1. (DOC 53 kb)

## Abbreviations

AKI: Acute kidney injury
APACHE: Acute Physiology and Chronic Health Evaluation
CKD: Chronic kidney disease
cTnI: Cardiac troponin I
cTnT: Cardiac troponin T
CVP: Central venous pressure
CV: Coefficient of variation
eGFR: Estimated glomerular filtration rate
ESRF: End-stage renal failure
ICU: Intensive care unit
IHD: Ischaemic heart disease
IQR: Interquartile range
KDIGO: Kidney Disease Improving Global Outcomes
MDRD: Modification of Diet in Renal Disease
MI: Myocardial infarction
NT-proBNP: N-terminal pro-brain natriuretic peptide
OR: Odds ratio
REC: Research Ethics Committee
RRT: Renal replacement therapy
SD: Standard deviation
SOFA: Serial Organ Failure Assessment
