# Supplementary material for: Cardiac biomarkers are associated with maximum stage of acute kidney injury in critically ill patients: a prospective analysis
Source: Crit Care. 2017 Apr 12;21:88. doi: 10.1186/s13054-017-1674-5 (PMC5388994; doi:10.1186/s13054-017-1674-5)
Supplement: Additional file 1: — Is a table presenting associations between cTnI, cTnT and NT-proBNP and the odds of AKI – excluding patients with any degree of AKI on day 1. (DOC 53 kb) [file 13054_2017_1674_MOESM1_ESM.doc]

**Additional file 1. Associations between cardiac troponin I, cardiac troponin T and NT-proBNP and odds of maximum stage of AKI – excluding patients with any AKI on day 1**

|  | **Total** | | **Median (IQR)** | **Median (IQR)** | **OR* (95% CI)** | **p-value** |
| --- | --- | --- | --- | --- | --- | --- |
|  | (1) | (2) | (1) | (2) |  |  |
| **Cardiac Troponin I** |  |  |  |  |  |  |
| Any AKI (1) vs no AKI (2) | 12 | 65 | 0.09  (0.04 - 0.28) | 0.02  (0.02 - 0.06) | 1.16  (0.92 - 1.46) | 0.208 |
| Stage 3 AKI (1) vs lower stage (2) | 7 | 70 | 0.23  (0.04 - 1.70) | 0.03  (0.02 - 0.12) | 1.26  (0.96 - 1.94) | 0.091 |
| Stage 3 AKI with RRT (1) vs lower stage (2) | 6 | 71 | 0.14  (0.04 to 0.33) | 0.03  (0.02 to 0.14) | 1.17  (0.87 to 1.56) | 0.296 |
| **Cardiac Troponin T** |  |  |  |  |  |  |
| Any AKI (1) vs no AKI (2) | 12 | 65 | 0.05  (0.02 - 0.09) | 0.02  (0.01 - 0.05) | 1.19  (0.90 - 1.57) | 0.215 |
| Stage 3 AKI (1) vs lower stage (2) | 7 | 70 | 0.07  (0.01 - 0.05) | 0.03  (0.01 - 0.05) | 1.30  (0.93 - 1.84) | 0.129 |
| Stage 3 AKI with RRT (1) vs lower stage (2) | 6 | 71 | 0.06  (0.02 - 0.12) | 0.03  (0.01 - 0.06) | 1.26  (0.88 - 1.81) | 0.212 |
| **NT-proBNP** |  |  |  |  |  |  |
| Any AKI (1) vs no AKI (2) | 12 | 65 | 6457  (2517 - 19895) | 1543  (239 - 5301) | 1.37  (1.05 - 1.80) | 0.021 |
| Stage 3 AKI (1) vs lower stage (2) | 7 | 70 | 11018  (2504 - 53270) | 1557  (239 - 5373) | 1.70  (1.12 - 2.59) | 0.013 |
| Stage 3 AKI with RRT (1) vs lower stage (2) | 6 | 71 | 11763  (2504 - 53270) | 1563  (239 - 6552) | 1.67  (1.08 - 2.58) | 0.021 |

Abbreviations: AKI = acute kidney injury; RRT = renal replacement therapy; IQR = interquartile range; CI = confidence interval; NT-proBNP = N-terminal – pro brain natriuretic peptide; OR = odds ratio

*Odds ratios (OR) estimated using logistic regression models with troponin levels log_2_ transformed. ORs represent the change in odds of AKI associated with a doubling of troponin levels.
